# Are Deaf College Students More Sensitive to Unfair Information? Evidence from an ERP Study

**DOI:** 10.3390/brainsci14080788

**Published:** 2024-08-02

**Authors:** Le Sun, Qi Dong, Xue Du, Dongtao Wei

**Affiliations:** 1 College of Educational Science, Chongqing Normal University, Chongqing 401331, China; 2022210503206@stu.cqnu.edu.cn (L.S.); 2022210503201@stu.cqnu.edu.cn (Q.D.); 2 Key Laboratory of Cognition and Personality, Ministry of Education, Southwest University, Chongqing 400715, China

**Keywords:** fairness decision, deaf college students, ultimatum game, unfair information, ERP

## Abstract

To better understand the individual differences in fairness, we used event-related potentials (ERPs) to explore the fairness characteristics of deaf college students through the ultimatum game task. Behaviorally, the significant main effect of the proposal type was found, which meant both deaf and hearing college students showed a lower acceptance rate for the more unfair proposal. Interestingly, we found a significant interaction between group and proposal type in the early stage (N1). Moreover, in the deaf college group, N1 (induced by moderately and very unfair proposals) was significantly larger than that of fair proposals. However, we found that deaf college students had smaller amplitudes on P2 and P3 than hearing college students. These results suggested that deaf college students might pursue more equity strongly so they are more sensitive to unfair information in the early stage. In a word, we should provide more fair allocations for deaf college students in our harmonious society.

## 1. Introduction

A sense of fairness is an important determinant of human social decisions. Especially in our highly complex social environments, people must consider the consequences of their actions with others in social interactions, which influence the decision-making process [1]. As the proverb goes, “It is not the lack of wealth that is worrying, but the inequality of wealth”. Fairness norms play a vital role in social decision making.

The ultimatum game (UG) is widely used to examine individuals’ responses to fairness in resource allocation [2,3,4]. In the classic UG, two players divide a sum of money. The proposer decides how to divide the money, while the responder decides to accept or reject the proposal. If the responder accepts, the money is divided as proposed. If the responder rejects, both players receive nothing [5,6]. Multiple studies that have used UG have found that many responders reject unequal proposals [7,8,9]. The behavior of rejecting inequality is a social preference behavior in which individuals give up their interests in pursuit of fairness [10]. When treated unfairly, many people are willing to sacrifice their interests to punish unfair behaviors in others [11,12].

Previous studies have revealed that N1, P2, and P3 are the most often studied components involved in fair decision-making processing by using the UG task [13,14,15]. As we know, N1 and the P2 are early components, reflecting the early automated processing process [16,17]. The anterior N1 is a negative wave that appears about 100 ms after the visual stimulus is presented and reaches its peak at about 120 ms [18,19]. It is different to the posterior N1 (or N170) which is also evoked by visual stimulation and reaches its maximum at the temporal–occipital electrodes [20]. The anterior N1 component is affected by attention, and attended stimuli evoke a larger N1 than unattended stimuli [21], so novel or threatening stimuli induce more negative N1 [21,22,23,24,25]; the posterior N1 is thought to reflect visual discrimination processing [26]. Like N1, P2 is also related to early attention allocation [18,27]. The P2 component is a positive component that reaches its peak about 200 ms after stimulus presentation and usually appears in the frontal and central regions [17]. Salience stimuli can prompt individuals to allocate more attention resources to them, thereby inducing a larger P2 amplitude [28]. For example, fair proposals induced larger P2 amplitudes than unfair proposals, reflecting the individual’s intrinsic goal and motivation to pursue fairness [13,29]. The later cognitive evaluation stage of fairness processing is represented by P3. P3 is a centro-parietal positivity occurring within the 300–600 ms time window [30], which is related to the selective allocation of attention resources and the elaboration of processing [13,31]. P3 is affected by outcome valence and outcome size, and positive feedback and greater rewards induce greater P3 [32]. In the UG task, fair proposals induce larger P3 fluctuations than unfair proposals [15,29].

A great deal of research on fair decision making [13,33,34] focused on fairly homogeneous samples [35,36], consisting mainly of Western Caucasian children without any disabilities [37]. Examining a more diverse population can provide insights into the development of inequity aversion [38]. Previous research on equity sensitivity has focused more on people with mental illnesses, such as Alzheimer’s disease [39], depression [40], and anxiety [41]. Unlike mental illness, hearing loss in deaf people is an organic disease. Due to the specificity of organ diseases, hearing loss limits their verbal communication and prevents them from fully connecting with the rest of society, resulting in their relative particularity in social and psychological development [42].

According to the World Health Organization, 1.5 billion people worldwide suffer from hearing loss of varying severity, of which about 430 million people need hearing loss rehabilitation services, and there are 34 million children with hearing loss alone [43]. Early studies of deaf people found that the majority of deaf babies (90%) are born to hearing parents who typically do not have experience with deafness as a disability [44,45]. These children of hearing or non-signing parents are at risk for hindered language, communication, and socio-emotional development [46,47,48,49,50,51]. Previous results have revealed that the different linguistic environments [49,50,51], family [52,53,54], and social interactions experienced by deaf people [37,55] make deaf people have deficiencies in practical communication skills [56] and social-information-processing skills [57] compared to their hearing peers, which may affect how they perceive the fairness of decision making [58,59].

In terms of emotions, deaf college students have hearing impairments and cannot communicate smoothly with others. They may not be able to develop social emotions well in limited communication, resulting in more negative personal emotions and a tendency to depression compared to hearing college students [60]. In life and study, deaf college students tend to restrain themselves when encountering problems and do not let their feelings flow naturally, resulting in the inability to vent negative emotions. Over time, they are prone to somatization, anxiety, depression, and other symptoms [61]. From a cultural perspective, sign language and oral language are two oral systems, but they develop in the same Chinese social environment, which makes the deaf culture and hearing culture both contradictory and unified [62]. Compared with other cultures, deaf culture has very different characteristics. Deaf college students under the influence of this intertwined culture are bound to have different psychological characteristics.

Deaf college students not only suffer from varying degrees of physical and mental distress but also face many injustices in social life [63,64,65]. At the same time, how to promote the development of inclusive education is inseparable from the discussion of special education majors in colleges and universities. Deaf college students are the main population of special education in colleges and universities and are a special and important group among college students. Studying their perception and characteristics of fairness will help to gain a deeper understanding of the deaf college student group and provide support for the development of inclusive education in colleges and universities.

In summary, this study adopts the UG paradigm and ERP technology to study the fair decision-making characteristics of deaf college students and explores whether there are differences in the fair decision-making characteristics of deaf college students and hearing college students. Are deaf college students more sensitive to unfair information?

## 2. Methods

### 2.1. Participants

We initially recruited 41 hearing college students from Chongqing Normal University, but technical problems during the experiment resulted in incomplete data collection for 8 hearing college students. A total of 62 college students including 33 hearing college students (M = 19.51 years, SD = 1.09), 15 males, and 29 deaf college students (M = 21.34 years, SD = 1.42), 13 males, participated in our study. All participants were paid a base fee of CNY 30 (about USD 4.5), and the actual decision in the game task determined the final reward for each person.

All of the deaf college students were enrolled in the Department of Special Education of the Normal University. They enter the university through a single examination and a single enrollment for undergraduate education, enjoy the same educational resources as hearing college students, and also enjoy the qualification of a master’s degree.

All subjects were right-handed and had a typical self-reported mental status. They had typical visual acuity or corrected visual acuity, no color weakness or color blindness. All of the subjects had no prior knowledge of the economics gaming experiment. The deaf college students were all proficient in sign language and used it as their primary communication tool, while the hearing college students had no experience with sign language. All subjects signed an informed consent form. This experiment was approved by the Local Ethics Committee of Chongqing Normal University.

### 2.2. Experimental Design and Stimulation

THE experiment used a mixed design of 2 (group: deaf college student, hearing college student) × 3 (proposal type: fair, moderately unfair, very unfair), with 5/5 for the fair proposal, 6/4, and 7/3 for the moderately unfair proposal, 8/2 and 9/1 for the very unfair proposal [66]. The number before the slash is the proposer’s portion, and the number after the slash is the portion assigned to the responders. For example, 5/5 means a total of CNY 10, and the proposer and responder of the game may obtain the first and last amount, respectively. The dependent variables are the responder’s acceptance rate of each type of allocation offer and the EEG component when each allocation option was seen.

All deaf college students were responders, and they completed multiple rounds of ultimatum games. The computer program determined whether each round of the game resulted in an overall win (+CNY 10) or a loss (−CNY 10) and then presented the distribution plan proposed by the proposer to the responder, who decided whether to accept it. If the subject rejected the plan, both parties would receive CNY 0. If the plan was accepted, both parties would divide the money according to the plan. The subject’s final remuneration was increased or decreased according to the amount of remuneration he or she deserved for participating in the experiment and his or her choice in the game.

The experimental stimulus is gray Chinese characters or numbers on a black screen with the digital font Courier, size 36, allocated font for proposal and final result display. The pictures of the proposers are gray-scale pictures with 300 × 300 pixels. The figures are silhouettes to eliminate the influence of factors such as gender and facial attractiveness.

### 2.3. Procedures

Before the experiment began, the subjects carefully read the instructions which were explained to the deaf college student by a professional sign language interpreter and signed an informed consent form. They were told that they and another student would perform the experiment online at the same time but in different locations. However, the subject’s choice determines his final income as well as the final income of other students. Before the formal experiment, 20 rounds of practice were conducted to help players become familiar with the game rules and key operations.

**Figure 1:**
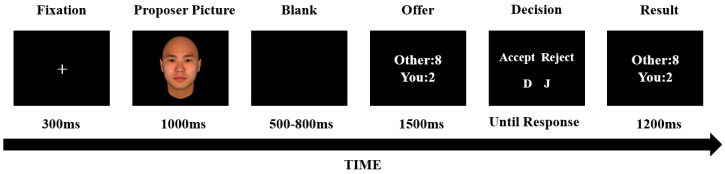
An example of the ultimatum game (UG).

At the beginning of each round of the game, a fixation point is presented in the center of the screen for 300 ms. Then, the game object of this round of game is presented, and the picture disappears after 1000 ms. After a black screen of 500–800 ms, the other party’s allocation proposal is presented for 1500 ms. During this period, the subject cannot make a key response. After the key prompt screen is presented, the subject responds with a key. After that, the result of this round of games is presented for 1200 ms. If the subject chooses to accept, the result is the same as the allocation proposal. If the subject chooses to reject, both parties receive 0. The appearance of the fixation point indicates the start of the next round of the game. The process of a single round of games and important controls are shown in Figure 1.

The experiment was conducted 180 times, with 60 repetitions for each condition, including 30 repetitions for each of the moderately unfair (including 6/4 and 7/3) and extremely unfair (including 8/2 and 9/1) proposals and 60 repetitions for 5/5. All experimental procedures were computer-controlled, and trials were arranged in a pseudo-random way to ensure that the same proposal type did not appear three times in a row.

### 2.4. EEG Recording and Analysis

The EEG was recorded from 64 electrode locations arranged in the standard 10–20 layout using Brain Vision Recorder software. During recording, the EEG data were referenced to the average voltage across channels, sampled at 1000 Hz, amplified (Brain Vision LLC, Morrisville, NC, USA), and filtered through a passband of DC ~280 Hz. Impedances were below 5 kΩ.

The EEG data were preprocessed offline and analyzed using Matrix Laboratory (MATLAB) R2016a (MathWorks, Natick, MA, USA) and EEGLAB 13.6.5b components. The analysis was performed using the mean value of both the papillae as a reference, with a filtered bandpass of 0.1–20 Hz. Artifacts of ±80 μV at all the electrodes were also excluded. The analysis time interval was from 200 ms before to 1000 ms after the presentation of the proposal type. We took the first 200 ms of the proposal type as the baseline. Trials with severe electromyogram (EMG) interference were excluded and eye movement artifacts were corrected by the independent component analysis (ICA) algorithm.

Based on visual observations of the grand average waveforms and the previous ERP studies [15,67], we averaged the ERP amplitude from the time range 110–150, 200–250, and 400–600 ms post-offer presentation for the N1, P2, and P3 analyses, respectively. According to the scalp distribution and previous reports, we selected nine electrode sites (Fz, F1, F2, FCz, FC1, FC2, Cz, C1, and C2) and six electrode sites (FCz, FC1, FC2, Cz, C1, and C2) in the frontal areas for the N1 and P2 analysis and nine electrode sites (Pz, P1, P2, POz, PO3, PO4, Oz, O1, and O2) in the central–parietal areas for P3 analysis.

The data were statistically analyzed using IBM SPSS Statistics 25.0. The acceptance rate of the subjects’ fair decision making was collected for behavioral data, and the mean wave amplitude of each component was selected for EEG data. A two-factor repeated-measures ANOVA of 2 (group: deaf college student, hearing college student) × 3 (proposal type: fair/moderately unfair/very unfair) was conducted for the acceptance rate and mean wave amplitude, respectively.

For all the analyses, the significance level was set at 0.05. Greenhouse–Geisser correction for ANOVA tests was used when appropriate. Post hoc comparisons were evaluated using the Newman–Keuls method. Partial eta-squared ($\eta_{p}^{2}$) values were provided to demonstrate effect size where appropriate, such that 0.05 represented a small effect, 0.10 represented a medium effect, and 0.20 represented a large effect [68].

## 3. Results

### 3.1. Behavior Results

The acceptance rates of deaf college students and hearing college students under different proposal types are shown in Table 1, and the ANOVA results under different proposal types are shown in Table 2.

The main effect of the proposal type for acceptance rate was significant, F(2, 59) = 502.36, *p* < 0.001, and $\eta_{p}^{2\;}$= 0.962, and the simple effect found that the acceptance rate of the fair proposal (0.98 ± 0.06%) was significantly greater than that of the moderately unfair proposal (0.54 ± 0.33%) and the very unfair proposal (0.10 ± 0.15%), and the moderately unfair proposal was significantly greater than that of the very unfair proposal. The moderately unfair proposal was significantly greater than the very unfair proposal.

### 3.2. EEG Results

**Figure 2:**
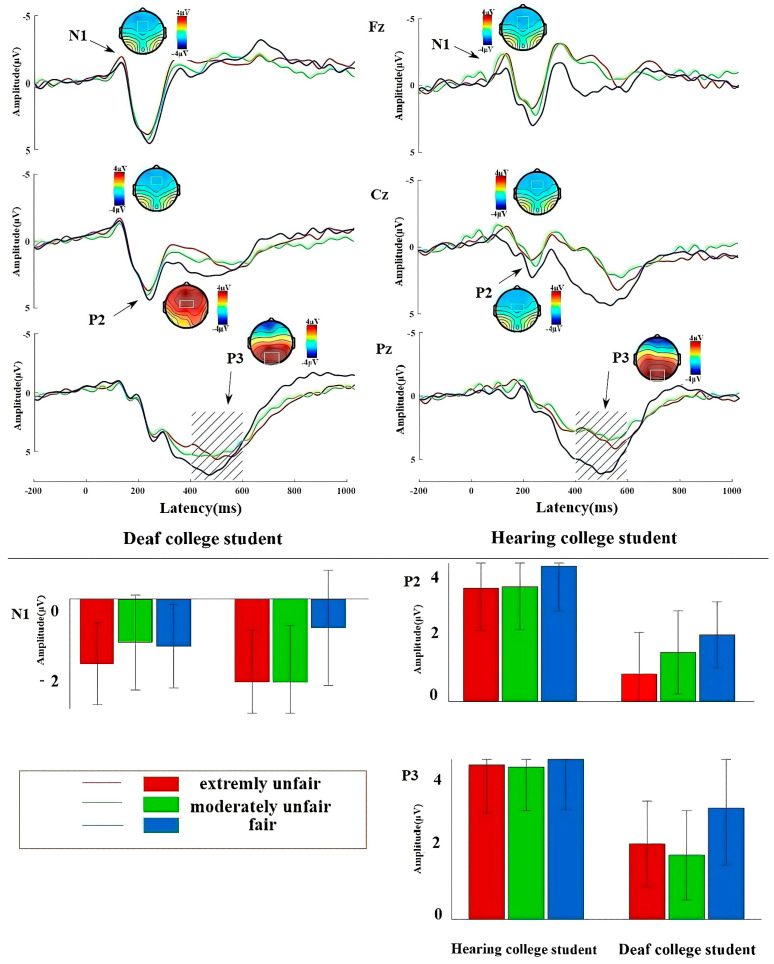
Waveforms, topography, and bar graphs of deaf and hearing college students under different proposal types. Note: Topographic waveforms and histograms of different groups, with topographic waveforms at the upper part and histograms at the lower part. The above picture shows the electroencephalogram (EEG), topographic map, and bar chart of deaf college students and hearing college students under different proposal types; the upper part of the black solid line is divided into a topographic map and waveform map, and the lower part is divided into bar charts; the red solid lines and bars represent extremely unfair conditions, the green solid lines and bars represent moderately unfair conditions, and the blue solid lines and bars represent fair conditions. The data in the bar graphs are represented by M ± SD.

The results of the analysis of variance for the ERP data are shown in Table 3, and the EEG waveforms, topographic maps, and bar graphs for each group are shown in Figure 2.

#### 3.2.1. N1

The main effect of proposal type was significant, F(2, 59) = 5.242, *p* = 0.008, $\eta_{p}^{2}$ = 0.151, and compared to the fair proposal (−0.72 ± 0.21 μV), the moderately unfair proposal (−1.20 ± 0.34 μV) and the very unfair proposal (−1.40 ± 0.40 μV) induced a more negative N1, *p* = 0.024 and *p* = 0.003. The interaction between the group and proposal level was significant, F(2, 59) = 3.546, *p* = 0.035, $\eta_{p}^{2}$ = 0.107, and simple effects analysis found that in the deaf college student group, the moderately unfair proposal (−1.58 ± 0.50 μV) induced a significantly greater mean wave amplitude than the fair proposal (−0.54 ± 0.48 μV), *p* < 0.001, and the very unfair proposal (−1.57 ± 0.44 μV) evoked a significantly greater mean wave amplitude than the fair proposal (−0.54 ± 0.48 μV), *p* = 0.002, F(2, 59) = 7.314, *p* = 0.001, $\eta_{p}^{2}$ = 0.199; there was no similar effect in the group of hearing college students.

#### 3.2.2. P2

The main effect of the group was significant (F(1, 60) = 7.460, *p* = 0.008, $\eta_{p}^{2}\;$= 0.111). The average amplitude induced by deaf college students (2.13 ± 0.45 μV) was significantly smaller than that of hearing college students (3.68 ± 0.52 μV). The main effect of the proposal level was significant (F(2, 59) = 5.336, *p* = 0.007, $\eta_{p}^{2}$ = 0.153). The average amplitude induced by extremely unfair proposals (2.13 ± 0.45 μV) was significantly smaller than that of fair proposals (3.07 ± 0.43 μV), *p* = 0.002. The average amplitude induced by moderately unfair proposals (2.49 ± 0.45 μV) was significantly smaller than that of fair proposals (3.07 ± 0.43 μV). The interaction between group and proposal type was not significant.

#### 3.2.3. P3

The main effect of the group was significant (F(1, 60) =5.154, *p* = 0.009, $\eta_{p}^{2}$ = 0.149). The average amplitude induced by deaf college students (2.19 ± 0.64 μV) was significantly smaller than that of hearing college students (4.20 ± 0.60 μV). The main effect of the proposal level was significant (F(2, 59) = 5.154, *p* = 0.009, $\eta_{p}^{2}$ = 0.149). The average amplitude induced by fair proposals (3.72 ± 0.55 μV) was significantly larger than that induced by extremely unfair proposals (3.02 ± 0.43 μV), *p* = 0.033, and moderately unfair proposals (2.84 ± 0.40 μV), *p* = 0.002. And the interaction of group and proposal type was not significant.

## 4. Discussion

This study explored whether deaf college students were more sensitive to unfair information than hearing college students in the UG task by using ERP. For the behavioral results, there is no difference in the rejection rate between deaf and hearing college students. In terms of ERP results, we found that deaf college students showed more negative N1 when faced with unfair and smaller P2 and P3.

In terms of behavioral outcomes, we found the significant main effect of the type of proposals. Higher levels of unfair proposals lead to lower acceptance rates among all participants, indicating a rejection of unfairness [12,69]. The findings support the fairness theory over the rational person hypothesis [70,71], which is that deaf college students display a tendency to reject inequality and punish others in pursuit of fairness, even when they do not directly benefit [11,12,72]. The lack of significant differences between groups may be attributed to similarities in the social–emotional and social–behavioral development of deaf and hearing college students. Previous behavioral studies have shown no disparities in social maturity between these two groups [73,74]. Deaf children and adolescents exhibit similar equity preferences under certain conditions compared to their hearing counterparts [37].

Traditional psychology primarily relies on behavioral response time and accuracy to study mental processes, which only allows for indirect speculation [75,76,77]. The development of cognitive neuroscience, which combines cognitive psychology and neuroscience techniques, directly uncovers the neural mechanisms of mental processes in the brain [78,79]. More and more research has focused on fairness by using ERP [80].

N1 as an early component reflects the allocation of attention to task-relevant stimuli before stimulus evaluation, and more threatening or novel stimuli induce more negative N1 [24,81,82]. In our study, we found an interaction between the proposal types and the group of N1. A further simple effect revealed that only in deaf college students, N1 caused by moderately and very unfair proposals was significantly higher than that of fair proposals [83]. On the one hand, as we know, attention to salient objects should also depend on the familiarity of stimuli, which strongly affects the visual attention of individuals [84]. Compared with hearing college students, deaf college students have inherent disadvantages in social interactions and are more sensitive to unfair events, causing them to devote more attention resources to unfair information (i.e., unfair proposals), whether it is moderately unfair or very unfair [85]. On the other hand, a large number of previous studies have shown that patients with anxiety disorders have an increased early processing of visual stimuli of threats (heightened alertness to threats) compared to healthy individuals [86,87,88]. Deaf college students may have interpreted the unfair bets as signals of social exclusion, thus feeling more anxious or worried about them [60]. Therefore, anxiety about the presence of unfair information in the deaf college student group may also cause a more negative N1 amplitude. At the same time, seminal ERP and neuroscience work on brain reorganization in deaf individuals has been carried out by Helen Neville and her collaborators (such as Daphne Bavelier), showing that deaf individuals undergo plastic changes in the brain pathways/areas involving the other sensory modalities. In particular, they have been shown to have better behavioral performance and related increased ERP/fMRI activity in visual attention and visual search tasks [89,90,91]. Therefore, in this study, deaf college students are more sensitive to unfair information, probably because of their better perceptual and attentional visual processing.

About 200 ms after the proposal was presented, the P2 amplitude induced by deaf college students was significantly smaller than that of hearing college students. The P2 component is generally considered to be an indicator of the perceptual analysis stage, reflecting the intensity of perceptual processing and the allocation of attentional resources [92]. Our result showed that as fairness processing proceeds, deaf college students invested less in the fairness decision-making task. Research has found that P2 is sensitive to the motivational information of stimuli, with larger amplitudes associated with outcomes that meet motivational goals [27,93]. Specifically, the P2 amplitude induced by unfair proposals was significantly smaller than that of fair proposals. Our result is consistent with previous research [29], reflecting the intrinsic goal and motivation of individuals to pursue fairness [13].

P3 represents late-stage cognitive processing [94]. A larger P3 means a greater investment of psychological resources and emotions, and the process of stimuli will be more refined and in-depth [15,95,96,97]. Therefore, in this study, the P3 amplitude induced by deaf college students is significantly smaller than that of hearing college students, which may indicate that deaf college students might invest less attention resources and emotional involvement [98]. Research has indicated that P3 is linked to attentional allocation and a high level of motivational or affective assessment processes, specifically in terms of subjective awareness and the importance of conveying desired outcomes [15,95,96,97], consistent with previous studies. Fair proposals in this study elicited a greater P3 response than unfair proposals, indicating that fair proposals were in line with individual fairness preferences [29,94,99].

To sum up, in the early stage of fair processing, compared with unfair proposals, the N1 amplitude induced by deaf college students is more negative when facing unfair proposals, indicating that deaf college students are more sensitive to unfair information. As the fairness processing time progresses, the P2 and P3 amplitudes induced by deaf college students are significantly smaller than those of hearing college students, indicating that deaf college students invest less in tasks and avoid more fair decisions.

Although we have provided some insights into equity for deaf people, this study still has several limitations. ERP technology has high temporal accuracy but poor spatial localization, while fMRI spatial localization is better. Therefore, future research should use both ERP and fMRI to study fair decision making in deaf people. Additionally, our sample size was small, so this may be a limitation of this study. Finally, another factor influencing ERP processing is the current emotional state, which was not collected in this study, which lacked emotional reporting. Therefore, subsequent studies of deaf college students should take into account their emotional state.

## 5. Conclusions

Overall, deaf college students were only more sensitive to unfair information in the early stage of decision making but may avoid being involved in anything decision-making-related.

## Figures and Tables

**Table 1:** Acceptance rates of deaf college students and hearing college students under different proposal types (M ± SD). Unit: %.

	Fair	Moderately Unfair	Very Unfair
deaf college students	0.98 ± 0.06	0.52 ± 0.32	0.09 ± 0.19
hearing college students	0.97 ± 0.06	0.59 ± 0.35	0.11 ± 0.12

**Table 2:** ANOVA results for deaf college students and hearing college students with different proposal types.

	Group			Proposal Type			Group × Proposal Type		
acceptance rate	F	*p*	$\eta_{p}^{2}$	F	*p*	$\eta_{p}^{2}$	F	*p*	$\eta_{p}^{2}$
	0.400	0.530	0.007	502.36	0.000	0.945	0.377	0.688	0.013

**Table 3:** ANOVA results of EEG data for deaf college students and hearing college students with different proposal types.

	Group			Proposal Type			Group × Proposal Type		
	F	*p*	$\eta_{p}^{2}$	F	*p*	$\eta_{p}^{2}$	F	*p*	$\eta_{p}^{2}$
N1	0.173	0.679	0.003	5.242	0.008	0.151	3.546	0.035	0.107
P2	7.460	0.008	0.111	5.336	0.007	0.153	0.692	0.505	0.023
P3	5.285	0.025	0.081	5.154	0.009	0.149	0.876	0.422	0.029

## Data Availability

The original contributions presented in the study are included in the article, and further inquiries can be directed to the corresponding authors.

## References

[1] Rilling J.K., Sanfey A.G. (2011). The neuroscience of social decision-making. Annu. Rev. Psychol..

[2] Güth W., Tietz R., Tietz R., Albers W., Selten R. (1988). Ultimatum Bargaining for a Shrinking Cake—An Experimental Analysis—. Proceedings of the Bounded Rational Behavior in Experimental Games and Markets Bielefeld.

[3] Kagel J.H., Kim C., Moser D. (1996). Fairness in Ultimatum Games with Asymmetric Information and Asymmetric Payoffs. Games Econ. Behav..

[4] Osumi T., Ohira H. (2009). Cardiac responses predict decisions: An investigation of the relation between orienting response and decisions in the ultimatum game. Int. J. Psychophysiol.

[5] Alexopoulos J., Pfabigan D., Lamm C., Herbert B., Fischmeister F. (2012). Do we care about the powerless third? An ERP study of the three-person ultimatum game. Front. Hum. Neurosci..

[6] Zheng L., Guo X., Zhu L., Li J., Chen L., Dienes Z. (2015). Whether others were treated equally affects neural responses to unfairness in the Ultimatum Game. Soc. Cogn. Affect. Neurosci..

[7] Li Q., Wang C., Taxer J., Yang Z., Zheng Y., Liu X. (2017). The Influence of Counterfactual Comparison on Fairness in Gain-Loss Contexts. Front. Psychol..

[8] Liao J., Ou J., Hu Y., Tobler P.N., Wu Y. (2023). Testosterone administration modulates inequality aversion in healthy males: Evidence from computational modeling. Psychoneuroendocrinology.

[9] Yang Z., Zheng Y., Wang C., Lai X., Hu K., Li Q., Liu X. (2022). Fairness decision-making of opportunity equity in gain and loss contexts. J. Exp. Soc. Psychol..

[10] Achtziger A., Alós-Ferrer C., Wagner A.K. (2016). The impact of self-control depletion on social preferences in the ultimatum game. J. Econ. Psychol..

[11] Decety J., Yoder K.J. (2017). The Emerging Social Neuroscience of Justice Motivation. Trends Cogn. Sci..

[12] Fehr E., Gächter S. (2002). Altruistic punishment in humans. Nature.

[13] Hu X., Mai X. (2021). Social value orientation modulates fairness processing during social decision-making: Evidence from behavior and brain potentials. Soc. Cogn. Affect. Neurosci..

[14] Polezzi D., Sartori G., Rumiati R., Vidotto G., Daum I. (2010). Brain correlates of risky decision-making. NeuroImage.

[15] Wu Y., Zhou X. (2009). The P300 and reward valence, magnitude, and expectancy in outcome evaluation. Brain Res..

[16] Eisenberger N. (2014). Social Pain and the Brain: Controversies, Questions, and Where to Go from Here. Annu. Rev. Psychol..

[17] Horat S.K., Herrmann F.R., Favre G., Terzis J., Debatisse D., Merlo M.C.G., Missonnier P. (2016). Assessment of mental workload: A new electrophysiological method based on intra-block averaging of ERP amplitudes. Neuropsychologia.

[18] Luck S.J., Hillyard S.A. (1994). Electrophysiological correlates of feature analysis during visual search. Psychophysiology.

[19] Wascher E., Hoffmann S., Sänger J., Grosjean M. (2009). Visuo-spatial processing and the N1 component of the ERP. Psychophysiology.

[20] Hillyard S.A., Anllo-Vento L. (1998). Event-related brain potentials in the study of visual selective attention. Proc. Natl. Acad. Sci. USA.

[21] Duzcu H., Özkurt T.E., Mapelli I., Hohenberger A. (2019). N1-P2: Neural markers of temporal expectation and response discrimination in interval timing. Acta. Neurobiol. Exp..

[22] Annic A., Bocquillon P., Bourriez J.-L., Derambure P., Dujardin K. (2014). Effects of stimulus-driven and goal-directed attention on prepulse inhibition of the cortical responses to an auditory pulse. Clin. Neurophysiol..

[23] Bouwer F.L., Honing H., Slagter H.A. (2020). Beat-based and Memory-based Temporal Expectations in Rhythm: Similar Perceptual Effects, Different Underlying Mechanisms. J. Cogn. Neurosci..

[24] Ito T.A., Bartholow B.D. (2009). The neural correlates of race. Trends Cogn. Sci..

[25] Kubota J.T., Ito T.A. (2007). Multiple cues in social perception: The time course of processing race and facial expression. J. Exp. Soc. Psychol..

[26] Vogel E.K., Luck S.J. (2000). The visual N1 component as an index of a discrimination process. Psychophysiology.

[27] Potts G.F., Patel S.H., Azzam P.N. (2004). Impact of instructed relevance on the visual ERP. Int. J. Psychophysiol..

[28] Carretié L., Hinojosa J.A., Martín-Loeches M., Mercado F., Tapia M. (2004). Automatic attention to emotional stimuli: Neural correlates. Hum. Brain Mapp..

[29] Wu Y., Leliveld M.C., Zhou X. (2011). Social distance modulates recipient’s fairness consideration in the dictator game: An ERP study. Biol. Psychol..

[30] Holroyd C.B., Coles M.G.H. (2002). The neural basis of human error processing: Reinforcement learning, dopamine, and the error-related negativity. Psychol. Rev..

[31] Yu R., Hu P., Zhang P. (2015). Social distance and anonymity modulate fairness consideration: An ERP study. Sci. Rep..

[32] Bellebaum C., Daum I. (2008). Learning-related changes in reward expectancy are reflected in the feedback-related negativity. Eur. J. Neurosci.

[33] Bianchi E.C., Brockner J., van den Bos K., Seifert M., Moon H., van Dijke M., De Cremer D. (2015). Trust in Decision-Making Authorities Dictates the Form of the Interactive Relationship Between Outcome Fairness and Procedural Fairness. Pers. Soc. Psychol. Bull..

[34] Osumi T., Ohira H. (2010). The positive side of psychopathy: Emotional detachment in psychopathy and rational decision-making in the ultimatum game. Personal. Individ. Differ..

[35] Price K.H., Lavelle J.J., Henley A.B., Cocchiara F.K., Buchanan F.R. (2006). Judging the fairness of voice-based participation across multiple and interrelated stages of decision making. Organ. Behav. Hum. Decis. Process..

[36] Radke S., Güroğlu B., de Bruijn E.R.A. (2012). There’s something about a fair split: Intentionality moderates context-based fairness considerations in social decision-making. PLoS ONE.

[37] Eichengreen A., Broekhof E., Güroğlu B., Rieffe C. (2020). Fairness decisions in children and early adolescents with and without hearing loss. Soc. Dev..

[38] Blake P.R., McAuliffe K., Corbit J., Callaghan T.C., Barry O., Bowie A., Kleutsch L., Kramer K.L., Ross E., Vongsachang H. (2015). The ontogeny of fairness in seven societies. Nature.

[39] Yerstein O., Carr A.R., Jimenez E., Mendez M.F. (2020). Neuropsychiatric Effects on Decision-Making in Early Alzheimer Disease. J. Geriatr. Psychiatry Neurol..

[40] Gradin V.B., Pérez A., MacFarlane J.A., Cavin I., Waiter G., Engelmann J., Dritschel B., Pomi A., Matthews K., Steele J.D. (2015). Abnormal brain responses to social fairness in depression: An fMRI study using the Ultimatum Game. Psychol. Med..

[41] Luo Y., Wu T., Broster L.S., Feng C., Zhang D., Gu R., Luo Y.-J. (2014). The temporal course of the influence of anxiety on fairness considerations. Psychophysiology.

[42] Witter M., de Rooij A., van Dartel M., Krahmer E. (2022). Bridging a sensory gap between deaf and hearing people–A plea for a situated design approach to sensory augmentation. Front. Comput. Sci..

[43] Chadha S., Kamenov K., Cieza A. (2021). The world report on hearing, 2021. Bull. World Health Organ..

[44] Jean Y.Q., Mazlan R., Ahmad M., Maamor N. (2018). Parenting Stress and Maternal Coherence: Mothers With Deaf or Hard-of-Hearing Children. Am. J. Audiol..

[45] Mitchell R.E., Karchmer M. (2004). Chasing the Mythical Ten Percent: Parental Hearing Status of Deaf and Hard of Hearing Students in the United States. Sign Lang. Stud.

[46] Hintermair M. (2006). Parental resources, parental stress, and socioemotional development of deaf and hard of hearing children. J. Deaf Stud. Deaf Educ..

[47] Howe D. (2006). Disabled Children, Maltreatment and Attachment. Br. J. Soc. Work..

[48] Howe D. (2006). Disabled children, parent-child interaction and attachment. Child Fam. Soc. Work..

[49] Humphries T., Kushalnagar P., Mathur G., Napoli D.J., Padden C., Rathmann C., Smith S.R. (2012). Language acquisition for deaf children: Reducing the harms of zero tolerance to the use of alternative approaches. Harm. Reduct. J..

[50] Peterson C., Slaughter V., Moore C., Wellman H.M. (2016). Peer social skills and theory of mind in children with autism, deafness, or typical development. Dev. Psychol..

[51] Sarant J.Z., Harris D.C., Galvin K.L., Bennet L.A., Canagasabey M., Busby P.A. (2018). Social Development in Children With Early Cochlear Implants: Normative Comparisons and Predictive Factors, Including Bilateral Implantation. Ear Hear..

[52] Oliva G. (2007). Looking Back... Longing for a Group of Friends. Odyssey: New Dir. Deaf. Educ..

[53] Punch R., Hyde M. (2011). Social participation of children and adolescents with cochlear implants: A qualitative analysis of parent, teacher, and child interviews. J. Deaf Stud. Deaf Educ..

[54] Zaidman-Zait A., Dotan A. (2017). Everyday Stressors in Deaf and Hard of Hearing Adolescents: The Role of Coping and Pragmatics. J. Deaf Stud. Deaf Educ..

[55] Rieffe C., Broekhof E., Eichengreen A., Kouwenberg M., Veiga G., da Silva B.M.S., van der Laan A., Frijns J.H.M. (2018). Friendship and Emotion Control in Pre-Adolescents With or Without Hearing Loss. J. Deaf Stud. Deaf Educ..

[56] Most T., Shina-August E., Meilijson S. (2010). Pragmatic abilities of children with hearing loss using cochlear implants or hearing AIDS compared to hearing children. J. Deaf Stud. Deaf Educ..

[57] Torres J., Saldaña D., Rodríguez-Ortiz I.R. (2016). Social Information Processing in Deaf Adolescents. J. Deaf Stud. Deaf Educ..

[58] Porter A., Creed P., Hood M., Ching T.Y.C. (2018). Parental Decision-Making and Deaf Children: A Systematic Literature Review. J. Deaf Stud. Deaf Educ..

[59] Singleton J.L., Tittle M.D. (2000). Deaf Parents and Their Hearing Children. J. Deaf Stud. Deaf Educ..

[60] Li Q., Ma X., Lu C. (2009). Characteristics and Relationship between Automatic Thinking and Dysfunctional Attitudes in Deaf College Students. Spec. Educ. China.

[61] Huang J., Lou X. (2006). A review of research on the mental health of deaf college students. Spec. Educ. China.

[62] Zhang S., Xu T. (2010). Study on Chinese Deaf Culture under the Influence of Western Deaf Culture. Spec. Educ. China.

[63] Anderson M.L., Leigh I.W. (2011). Intimate partner violence against deaf female college students. Violence Against Women.

[64] Luft P. (2000). Communication barriers for deaf employees: Needs assessment and problem-solving strategies. Work.

[65] Samar V.J., Parasnis I. (2007). Cortical locus of coherent motion deficits in deaf poor readers. Brain Cogn..

[66] Harlé K.M., Sanfey A.G. (2007). Incidental sadness biases social economic decisions in the Ultimatum Game. Emotion.

[67] Alexander R., Aragón O.R., Bookwala J., Cherbuin N., Gatt J.M., Kahrilas I.J., Kästner N., Lawrence A., Lowe L., Morrison R.G. (2021). The neuroscience of positive emotions and affect: Implications for cultivating happiness and wellbeing. Neurosci. Biobehav. Rev..

[68] Cohen S., Conduit R., Lockley S., Rajaratnam S., Cornish K. (2014). The relationship between sleep and behavior in autism spectrum disorder (ASD): A review. J. Neurodev. Disord..

[69] Gong Y., Yao L., Chen X., Xia Q., Jiang J., Du X. (2022). Group Membership Modulates Fairness Consideration Among Deaf College Students—An Event-Related Potential Study. Front. Psychol..

[70] Chang Y.-H., Levinboim T., Maheswaran R., Guy T.V., Kárný M., Wolpert D.H. (2012). The Social Ultimatum Game. Decision Making with Imperfect Decision Makers.

[71] Koenigs M., Tranel D. (2007). Irrational economic decision-making after ventromedial prefrontal damage: Evidence from the Ultimatum Game. J. Neurosci..

[72] Rand D.G., Tarnita C.E., Ohtsuki H., Nowak M.A. (2013). Evolution of fairness in the one-shot anonymous Ultimatum Game. Proc. Natl. Acad. Sci..

[73] Marschark M., Kronenberger W.G., Rosica M., Borgna G., Convertino C., Durkin A., Machmer E., Schmitz K.L. (2017). Social Maturity and Executive Function Among Deaf Learners. J. Deaf Stud. Deaf Educ..

[74] Marschark M., Walton D., Crowe K., Borgna G., Kronenberger W.G. (2018). Relations of Social Maturity, Executive Function, and Self-Efficacy Among Deaf University Students. Deaf. Educ. Int..

[75] Heitz R.P. (2014). The speed-accuracy tradeoff: History, physiology, methodology, and behavior. Front. Neurosci..

[76] Ratcliff R., Smith P.L., Brown S.D., McKoon G. (2016). Diffusion Decision Model: Current Issues and History. Trends Cogn. Sci..

[77] Wickelgren W.A. (1977). Speed-accuracy tradeoff and information processing dynamics. Acta Psychol..

[78] Arzi A., Banerjee S., Cox J., Souza D., De Brigard F., Doll B., Fairley J., Fleming S., Herholz S., King D. (2014). The Significance of Cognitive Neuroscience: Findings, Applications, and Challenges.

[79] Barack D.L., Krakauer J.W. (2021). Two views on the cognitive brain. Nat. Rev. Neurosci..

[80] Wu Y., Zhou X.-L. (2013). The Context-Dependency of Fairness Processing: Evidence from ERP Study: The Context-Dependency of Fairness Processing: Evidence from ERP Study. Acta Psychol. Sin..

[81] Clark V.P., Hillyard S.A. (1996). Spatial Selective Attention Affects Early Extrastriate But Not Striate Components of the Visual Evoked Potential. J. Cogn. Neurosci..

[82] Potts G.F. (2004). An ERP index of task relevance evaluation of visual stimuli. Brain Cogn..

[83] Ruiz-Martínez F.J., Morales-Ortiz M., Gómez C.M. (2022). Late N1 and postimperative negative variation analysis depending on the previous trial history in paradigms of increasing auditory complexity. J. Neurophysiol..

[84] Jurkat S., Köster M., Yovsi R., Kärtner J. (2020). The Development of Context-Sensitive Attention Across Cultures: The Impact of Stimulus Familiarity. Front. Psychol..

[85] Karampidis K., Trigoni A., Papadourakis G., Christofaki M., Escudeiro N., Cristea A.I., Troussas C. (2021). Difficulties and Disparities to Distance Learning During COVID-19 Period for Deaf Students–A Proposed Method to Eradicate Inequalities. Proceedings of the Intelligent Tutoring Systems.

[86] Bar-Haim Y., Lamy D., Glickman S. (2005). Attentional bias in anxiety: A behavioral and ERP study. Brain Cogn..

[87] Li W., Zinbarg R.E., Paller K.A. (2007). Trait anxiety modulates supraliminal and subliminal threat: Brain potential evidence for early and late processing influences. Cogn. Affect. Behav. Neurosci..

[88] Williams L.M., Kemp A.H., Felmingham K., Liddell B.J., Palmer D.M., Bryant R.A. (2007). Neural biases to covert and overt signals of fear: Dissociation by trait anxiety and depression. J. Cogn. Neurosci..

[89] Bavelier D., Tomann A., Hutton C., Mitchell T., Corina D., Liu G., Neville H. (2000). Visual attention to the periphery is enhanced in congenitally deaf individuals. .J Neurosci..

[90] Neville H.J., Lawson D. (1987). Attention to central and peripheral visual space in a movement detection task: An event-related potential and behavioral study. I. Normal hearing adults. Brain Res..

[91] Neville H.J., Schmidt A., Kutas M. (1983). Altered visual-evoked potentials in congenitally deaf adults. Brain Res.

[92] Fan J., Li W., Lin M., Li X., Deng X. (2023). Effects of mindfulness and fatigue on emotional processing: An event-related potentials study. Front. Behav. Neurosci..

[93] Boudreau C., McCubbins M.D., Coulson S. (2009). Knowing when to trust others: An ERP study of decision making after receiving information from unknown people. Soc. Cogn. Affect. Neurosci..

[94] Wang Y., Kuhlman D.M., Roberts K., Yuan B., Zhang Z., Zhang W., Simons R.F. (2017). Social value orientation modulates the FRN and P300 in the chicken game. Biol. Psychol..

[95] Mussel P., Hewig J., Weiß M. (2018). The reward-like nature of social cues that indicate successful altruistic punishment. Psychophysiology.

[96] Nieuwenhuis S., Aston-Jones G., Cohen J.D. (2005). Decision making, the P3, and the locus coeruleus-norepinephrine system. Psychol. Bull..

[97] Schupp H., Cuthbert B., Bradley M., Hillman C., Hamm A., Lang P. (2004). Brain processes in emotional perception: Motivated attention. Cogn. Emot..

[98] Zhang H., Ma H., Xu F., Liu Y., Shi Y. (2018). Fairness Preference in Ultimatum Game: A Perspective Based on Dual System Theory. Adv. Psychol. Sci..

[99] Qu C., Wang Y., Huang Y. (2013). Social exclusion modulates fairness consideration in the ultimatum game: An ERP study. Front. Hum. Neurosci..

